# Management of Ureteral Stricture Disease After Radiation Therapy for Pelvic Malignancies: A Retrospective, Multi-Institutional Analysis

**DOI:** 10.3390/cancers16213561

**Published:** 2024-10-22

**Authors:** Marco Carilli, Valerio Iacovelli, Marta Signoretti, Antonio Luigi Pastore, Franco Gaboardi, Giovannalberto Pini, Mario Falsaperla, Roberto Falabella, Pierluigi Bove

**Affiliations:** 1 Robotic and Minimally-Invasive Urology Unit, Azienda Ospedaliero Universitaria, Policlinico Tor Vergata, 00133 Rome, Italy; 2 Urology Unit, Department of Medico-Surgical Sciences and Biotechnologies, ICOT, Sapienza University of Rome, 04100 Latina, Italy; 3 Department of Urology, IRCCS Ospedale San Raffaele Turro, 20127 Milan, Italy; 4 Department of Urology, ARNAS Garibaldi Hospital, 95123 Catania, Italy; 5 Urology Unit, AO San Carlo, 85100 Potenza, Italy

**Keywords:** robotic surgery, ureteral stricture, radiation therapy

## Abstract

Objectives. A radiation-induced ureteral stricture (RIUS) is a challenging condition in urologic surgery, and the optimal surgical strategy is still a matter of debate. The aim of this study was to report post-operative outcomes of minimally invasive management of this condition from a multi-institutional cohort. Methods. Data of patients with diagnoses of RIUS at five referral robotic centers between January 2017 and December 2022 were retrospectively analyzed. Pre-, intra- and post-operative variables were collected. Recurrence was defined as the presence of flank pain combined with imaging findings for obstruction, and requiring further management. Univariate and multivariate logistic regression models were built to identify predictors of ureteral stricture recurrence. Results. Fifty-three patients with a diagnosis of an RIUS were included. Primary pelvic malignancy was mostly cervical (42%). In 72% of cases (mostly mid-proximal and short strictures) endourological management was attempted, which was deemed successful in 74% of patients at stent removal. The endourology success rate at a median follow-up of 12 months was 59%. Twenty-five patients (47%) were scheduled for robotic surgery. The reconstructive techniques most often performed were end-to-end anastomosis (44%) and ureteral reimplantation (52%). Three low-grade Clavien-Dindo post-operative complications (12%) occurred. Robotic surgery was successful in all cases except one. Considering the entire cohort, eight patients (15%) developed recurrence at a median follow-up of 5 months. All patients with recurrence were managed by permanent drainage. A statistically significant improvement in eGFR with respect to baseline was found at a median follow-up of 12 months (*p* = 0.007). The univariate logistic regression model identified ureteral stricture length >2 cm (OR 6.4, 95% C.I. 1.1–36.9, *p* = 0.04) and concomitant chemotherapy (OR 8.9, 95% C.I. 1.6–49.9, *p* = 0.01) as predictors of recurrence. At multivariate analysis concomitant chemotherapy was confirmed as an independent predictor of recurrence (OR 7.8, 95% C.I. 1.3–49.0, *p* = 0.03). Conclusions. Endourological management of an RIUS is reasonable for short and mid-proximal ureteral strictures, while robotics is required in almost all the remaining cases. Up to 15% of recurrence develops within 6 months. Re-do reconstructive surgery is rarely performed, even in referral centers.

## Simple Summary

Ureteral stricture disease is a rare but fearsome complication after radiation therapy for pelvic malignancies. Several minimally-invasive managements have been proposed: however, the optimal strategy is still a matter of debate, and available literature on this topic is rather heterogeneous. This multi-institutional retrospective analysis showed that an endourological attempt is reliable in case of a short, mid-proximal stricture, while robot-assisted reconstructive surgery seems to best fit with the remaining cases. Up to 15% of recurrences develops within 6 months. Regardless of treatment strategy, a significant improvement in eGFR was found during follow-up. Stricture length and concomitant chemotherapy at the time of radiation therapy were identified as predictors of recurrence. An adequate preoperative strategy is paramount, since stricture’s features and oncologic anamnesis could be potential predictors of surgical failure.

## 1. Introduction

Radiogenic injuries of the ureter are quite rare, considering the relatively large number of patients with pelvic malignancies receiving radiation therapy [1,2,3]. Radiation techniques have advanced significantly during the past 30 years, with several improvements both in treatment planning and delivery systems [4]. However, despite the fact that these improvements have led to a reduction in side effects, a non-negligible proportion of patients develop urinary toxicity, including radiation-induced ureteral stricture (RIUS) disease [5,6].

An RIUS is a challenging condition in urologic surgery. Several minimally invasive managements have been proposed, ranging from endourologic procedures to open or robot-assisted reconstructive techniques [7]. It is extremely debated which strategy is the most effective in these patients. Historically, endourologic management of strictures > 2 cm, with mid-ureteral location, and/or secondary to radiation or ischemic injury, is associated with poor outcomes [8]. Currently, the widespread dissemination of robotic surgical platforms has made reconstructive surgery much more accessible in such tedious situations [9,10].

Given the paucity of data regarding the treatment of RIUSs, we herein present our multi-institutional analysis of patients who underwent minimally invasive management of this condition, aiming to report post-operative outcomes.

## 2. Materials and Methods

### 2.1. Patients

Data of patients with a diagnosis of an RIUS at 5 referral robotic centers (≥100 procedures/year) between January 2017 and December 2022 were retrospectively analyzed. We included in the study only patients who underwent external beam radiation therapy (EBRT) with standard fractionation. Patients scheduled for brachytherapy, hypofractionation, or stereotactic body radiation therapy (SBRT) were excluded, as well as patients with preoperative imaging that was not available or incomplete.

The study was conducted in accordance with the Declaration of Helsinki and its later amendments, and approved by the local institutional ethics committee.

### 2.2. Study Endpoint and Definition of Recurrence

The primary endpoint was a recurrence free-rate after the minimally invasive management of an RIUS.

Recurrence was defined as the presence of flank pain combined with imaging findings for obstruction, and requiring further management.

### 2.3. Outcomes Measurements

Patients with an RIUS were diagnosed by the history, laboratory tests, and upper urinary tract imaging, including at least a computed tomography (CT) urography, or a high-quality ureterogram (antegrade and/or retrograde). To classify the stricture’s site, the ureter was divided into proximal (between the uretero-pelvic junction and the upper margin of the sacrum), mid (between the upper and lower margins of the sacrum), and distal (between the lower margin of the sacrum and the ureteral orifice) sections at preoperative imaging.

Preoperative variables, including age, sex, body mass index (BMI), anatomical features of ureteral stricture (side, location, and length), history of previous endourological procedures (yes/no), baseline renal function assessment by serum creatinine and estimated glomerular filtration rate (eGFR) calculated using the Modification of Diet in Renal Disease equation [11], surgical procedures for primary pelvic malignancies, radiation therapy protocols (total dose, daily dose, and duration), time-to-diagnosis of the RIUS (i.e., the time between the end of radiotherapy and the onset of symptoms) and its immediate management were collected at baseline.

Perioperative data were collected, including endourologic and/or robotic reconstructive techniques, the length of hospital stay, and the renal function assessment at discharge. Eventual intra- and post-operative complications with or without readmissions (occurring within 3 months) were recorded and classified according to the Clavien-Dindo system [12].

Follow-up data were recorded as well, including a renal function assessment at the last follow-up visit, eventual ureteral stricture recurrence (yes/no), and the time-to-recurrence.

### 2.4. Surgical Techniques

Endoscopic procedures included retrograde balloon dilatation under fluoroscopy guidance or laser endoureterotomy. Dilatation was performed mainly by a 6- or 7-F, 4 to 6 cm balloon at 8 atmospheres; rarely, in cases of hard and/or tight strictures, a 15-F, 4 cm balloon at 20 atmospheres was used. Ho:YAG laser endoureterotomy was performed with 1–2 J and 10–20 Hz settings, depending on the cases [13,14].

All robotic procedures were performed by a da Vinci (Si, X or Xi) surgical system (Intuitive Surgical, Sunnyvale, CA, USA). Reconstructive techniques included end-to-end anastomosis, ureteral reimplantation (with or without a psoas hitch or Boari flap), and ureteral replacement with an ileal segment, depending on the case [15]. If necessary, intra-ureteral and/or intra-venous near-infrared fluorescence (NIRF) imaging was used [16].

After all procedures, a 6-F double-J ureteral stent was placed.

### 2.5. Statistical Analysis

Continuous variables were summarized using medians and interquartile ranges (IQR); frequencies and proportions were used to report categorical variables. All data were tested for normality using the Shapiro–Wilk test. Wilcoxon and Mann–Whitney non-parametric tests were performed to compare repeated measurements and independent samples, respectively. Fisher’s exact test was used for categorical variables. Univariate and multivariate logistic regression models were built to identify predictors of ureteral stricture recurrence. Data analysis was conducted using SPSS 21.0 software (IBM, Armonk, NY, USA). Statistical significance was defined as a *p*-value < 0.05.

## 3. Results

**Figure 1:**
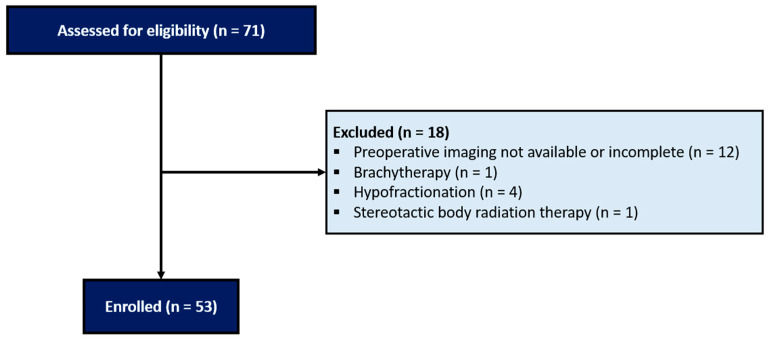
CONSORT flow diagram.

After accounting for the exclusion criteria (Figure 1), 53 patients with a diagnosis of an RIUS were included in the present analysis. Table 1 shows the distribution of baseline patients’ characteristics. No patient underwent previous endourological procedures. Primary pelvic malignancy was mostly cervical (42%). According to the lesion sites at preoperative imaging, ureteral strictures were classified as proximal (36%), mid (39%) or distal (25%). Most cases (64%) were short strictures (≤ 2 cm). Ureteral stenting was the most used initial management (68%).

In 38 patients (72%), endourological management was attempted. Specifically, 23 patients (61%) underwent balloon dilatation, while the other 15 patients (39%) underwent a laser endoureterotomy. Of note, the majority of patients managing their RIUS by endourology had proximal or mid-ureteral strictures (87%), and had a length ≤2 cm (74%). No patient developed intra- or post-operative complications. Endourological procedures were deemed successful in 28 out of 38 patients (74%) at stent removal (within 3 months), while the remaining 10 patients where scheduled for robotic reconstructive surgery due to persistence of symptoms. At a median follow-up of 12 months (IQR 12–24), six patients initially indicated as having successfully developed a stricture recurrence. The median time-to-recurrence in these patients was 4 months (IQR 3–7). None of these patients were scheduled for re-do endourological procedures or robotic surgery, due to severe comorbidities, age, or refusal; accordingly, all these patients finally underwent permanent drainage by ureteral stenting or nephrostomy. Overall, the endourological procedures’ success rate was 59%.

Twenty-five patients (47%) were scheduled for robotic surgery (60% immediate vs. 40% after endourology failure, as stated above). Reconstructive techniques most often performed were end-to-end anastomosis (44%) and ureteral reimplantation (52%). Adjunctive procedures (such as a psoas hitch or Boari flap) were undertaken in most of the reimplantations (77%) in order to achieve a tension-free anastomosis. Ureteral replacement with an ileal segment was performed in one case of a long (>4 cm) mid-ureteral stricture. All patients underwent stent removal within 3 months. Three low-grade (≤II) Clavien-Dindo post-operative complications (12%) occurred: two patients had transient ileus, while another patient (who underwent reimplantation plus a Boari flap) developed a peri-vesical urinary leakage, managed conservatively with prolonged catheterization (20 days). At a median follow-up of 12 months (IQR 12–12), robotic surgery was successful in all cases except one of a mid-ureteral stricture, in which a recurrence occurred after 8 months: in this case, a re-do end-to-end anastomosis was attempted unsuccessfully, so the patient required permanent stent positioning. Table 2 summarizes peri- and post-operative outcomes.

**Figure 2:**
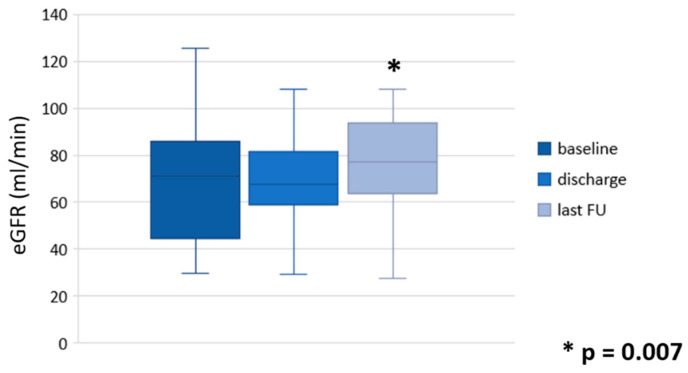
Changes in estimated glomerular filtration rate (eGFR, mL/min) during follow-up.

Among the entire cohort, no statistically significant difference in median eGFR was found between baseline and discharge (71.2 vs. 67.7 mL/min, *p* = 0.2), while a statistically significant improvement was found at a median follow-up of 12 months (67.7 vs. 77.0 mL/min, *p* = 0.007) (Figure 2). At the last follow-up visit, the median eGFR showed no statistically significant difference between patients who underwent endourologic and robotic management (*p* = 0.1).

Univariate logistic regression model identified a ureteral stricture length >2 cm (odds ratio [OR] 6.4, 95% C.I. 1.1–36.9, *p* = 0.04) and concomitant chemotherapy (OR 8.9, 95% C.I. 1.6–49.9, *p* = 0.01) as predictors of ureteral stricture recurrence. At multivariate analysis, concomitant chemotherapy was confirmed as an independent predictor of recurrence (OR 7.8, 95% C.I. 1.3–49.0, *p* = 0.03) (Table 3).

## 4. Discussion

The association between radiation therapy for pelvic malignancies and ureteral stricture disease is not new: indeed, an RIUS has been recognized as a complication of radiation therapy since 1920 [17]. Nonetheless, the available literature on this topic is rather heterogeneous, especially due to the rarity of this condition (overall incidence between 0.4 and 2.7%) [18]. Moreover, the management of an RIUS is complicated by several factors, such as the long average latency period (up to 20 years) [19] and the absence of symptoms in most cases [20].

Results from our study showed that the majority of cases (64%) are short strictures (≤2 cm). The time-to-diagnosis is about 6 months, and generally is managed by immediate stenting, followed by treatment with curative intent. In 72% of cases, there was an endourological attempt, with an overall success rate of 59% at a median follow-up of 12 months. In 47% of cases, a robotic reconstructive technique was necessary. Up to 15% of recurrence develops within 6 months. Regardless of treatment strategy, a significant improvement in eGFR was found during follow-up. Finally, long strictures (>2 cm) and concomitant chemotherapy at the time of radiation therapy were identified as predictors of stricture recurrence.

These results require several considerations. First, an endourological management seems reliable in the case of a short, mid-proximal stricture. In our cohort, 40 out of 53 patients (75%) had proximal or mid-ureteral strictures, and the majority of them were managed by endourology. It is interesting to note that, despite this case series being focused on RIUSs after pelvic malignancy treatments, there is an high percentage of mid-proximal strictures, which theoretically should be outside the irradiation field: however, considering that most malignancies were cervical (42%) or colo-rectal (28%), this percentage could be related to extended-fields radiotherapies, which are related to damages to upper abdominal structures [21,22]. Regardless of the strictures’ location and etiology, results from a retrospective analysis of endourological management for ureteral strictures (benign or post-malignant) showed an overall success rate of 72% [23]. Our results are slightly worse (a success rate of 59%), although we should take into account that we analyzed only post-malignant cases. It is reasonable to state that experienced robotic surgeons involved in this study feel themselves more comfortable with reconstructive surgery in cases of distal diseases, in which the ureter has likely been damaged not only by radiation therapy, but also by surgical procedures (e.g., radical prostatectomy, hysterectomy, etc.).

As extensively described in the literature, radiation toxicity is classified as acute (within days or weeks) or late (months or even years); some authors consider 90 days as the threshold between acute and late complications [7]. The median time-to-diagnosis in this study was 6 months (IQR 4–10), suggesting that irreversible structural changes in the ureteral wall are established early after radiation therapy. Radiation-induced ischemia leads to fibrosis and contraction in the ureter and, as a result, ureter stenosis develops [24]. Those structural changes also explain why the reconstructive surgery of an RIUS is technically challenging: as a matter of fact, re-do surgical procedures are rarely performed, even in referral centers; moreover, no patient in our cohort was scheduled for graft procedures (e.g., buccal mucosal ureteroplasty or appendiceal substitution), probably due to the fear of complications and/or failure. Rather, the majority of patients required procedures providing minimal ureteral dissection (to preserve the ureteral microvascular blood supply) and tension-free anastomosis (to lower recurrent disease) in such “low quality” ureters. In this sense, robotic end-to-end anastomosis or ureteral reimplantation (with or without adjunctive procedures) seem to be the best candidates, thanks to the intrinsic features of robotic platforms (e.g., magnified 3D vision, the eventual use of NIRF imaging, and 7-degrees of freedom instruments ensuring meticulous muco-mucosal sutures) [25].

The median overall time-to-recurrence was 5 months (IQR 3–8), and most of recurrences were described after endourologic management (only one case of robotic surgery failure). In the retrospective analysis by Reus and Brehmer, the recurrence rate after endourology was 17%, and all recurrences occurred in the post-malignant setting; moreover, the majority of recurrences were initially >2 cm and occurred within the first 12 months [23]. Our results are comparable, and the logistic regression model confirms the role of stricture length as a predictor of recurrence (*p* = 0.04).

Multivariate analysis showed that concomitant chemotherapy is a significant independent predictor of stricture recurrence (*p* = 0.03). As is well known, chemotherapy is generally associated with higher stage of disease, which is also associated with extended-fields radiotherapies, as already stated above. Due to the retrospective nature of this study, a complete pathological staging of every single patient was not available: however, locally advanced disease has been described as a risk factor for an RIUS [26], and it is plausible that this condition is prone to be “refractory” (at least to endoscopic procedures). Moreover, most of pelvic cancers arise in epithelial mucosa (e.g., the cervix, colon, prostate, bladder, etc.), and first-line chemotherapy often includes platinum-based regimens, which are associated with mucosal toxicity (especially oral or gastro-intestinal) [27]. Despite the fact that these drugs are not strictly associated with urothelial toxicity (such as cyclophosphamide and ifosfamide [28]), it is reasonable to assume that, in ureters already “compromised” by demolitive surgery and radiation therapy, the concomitant use of them may have a “synergistic effect”, possibly explaining the role of chemotherapy as a predictive factor of stricture recurrence.

Finally, this study highlighted that, in terms of post-operative eGFR, a significant benefit (*p* = 0.007) is not immediate, but after a few months. As shown in Table 1, 44 out of 53 patients (83%) had unilateral disease; perhaps, an earlier benefit would have been visible in patients with bilateral disease, in which, however, several predictors of renal function recovery after relief from ureteral obstruction have been described, with conflicting results [29,30]. Anyway, the small sample size did not allow us to carry out any reliable sub-analysis in this sense.

We acknowledge several limitations of this study. First, the retrospective nature of this study. Second, our results could be not fully generalized for several reasons: the heterogenous population treated (different pelvic malignancies with specific therapeutic protocols), the various surgical techniques used, and the number of the surgeons involved (although all of them had consolidated experience in the minimally invasive management of upper urinary tract obstruction). To mitigate the sample’s heterogeneity, we included only patients scheduled for EBRT, which represents the most common form of radiation therapy. Generally, pelvic malignancies received standard (Intensity Modulated Radiation Therapy, IMRT) or advanced (Volumetric Modulated Arc Therapy, VMAT) protocols [31]. Regardless of the EBRT technology, the clinical target volume included pelvic lymph node and presacral regions. Third, the interpretation of results may be influenced by the heterogeneity of follow-up protocols, which can vary among institutions in terms of frequency and imaging modality. Finally, we did not record the MAG3 renography to assess the degree of ureter stenosis and the function of the affected kidney, neither pre- nor post-operatively, since these data were not available for all the patients. Doubtless, renal scans would have provided even more detailed information about the functional recovery of every single renal unit, and in a real-life scenario, we strongly recommend to perform it before any surgical planning; however, since eGFR showed a significant improvement during follow-up, we can consider these data sufficient for a functional evaluation in our cohort analysis.

Notwithstanding these limitations, the results of this study contribute to expanding the current knowledge on the management of RIUSs. Although our study does not allow us to establish any strong conclusion, we underline the paramount importance of an adequate preoperative strategy, since strictures’ features and oncologic anamnesis could be potential predictors of surgical failure.

## 5. Conclusions

This multi-institutional analysis showed that the endourological management of an RIUS is reasonable for short and mid-proximal ureteral strictures, while reconstructive surgery is required in almost all the remaining cases. Up to 15% of recurrence develops within 6 months. Re-do reconstructive surgery is rarely performed, even in referral centers.

## Figures and Tables

**Table 1:** Clinical and demographic baseline patients’ characteristics.

	N = 53
Age (years), median (IQR)	62 (55–68)
BMI (kg/m^2^), median (IQR)	25.9 (23.9–28.3)
Ureteral stricture side, n (%)	
Right	19 (35.8)
Left	25 (47.2)
Bilateral	9 (17.0)
Ureteral stricture location, n (%)	
Proximal	19 (35.8)
Mid	21 (39.6)
Distal	13 (24.6)
Ureteral stricture length, n (%)	
≤2 cm	34 (64.1)
>2 cm	19 (35.9)
Pre-radiotherapy serum creatinine (mg/dL), median (IQR)	1.11 (0.92–1.40)
Pre-radiotherapy eGFR (mL/min), median (IQR)	71.2 (44.5–86.0)
Primary pelvic malignancy, n (%)	
Prostate	12 (22.6)
Cervical	22 (41.5)
Colo-rectal	15 (28.4)
Other	4 (7.5)
Total dose (Gy), median (IQR)	48 (45–63)
Daily dose (Gy/day), median (IQR)	2.0 (1.8–2.0)
Radiotherapy duration (weeks), median (IQR)	5 (5–5)
Concomitant chemotherapy, n (%)	
No	43 (81.1)
Yes	10 (18.9)
Time-to-diagnosis (months), median (IQR)	6 (4–10)
Immediate management at diagnosis, n (%)	
Ureteral stenting	36 (67.9)
Nephrostomy	17 (32.1)

IQR, interquartile range; BMI, Body Mass Index; eGFR, estimated glomerular filtration rate.

**Table 2:** Peri- and post-operative outcomes.

	N = 53
Endourological management, n (%)	
No attempt	15 (28.3)
Proximal	17 (32.1)
Mid	16 (30.2)
Distal	5 (9.4)
Robotic reconstructive technique, n (%)	
End-to-end anastomosis	11 (44.0)
Ureteral reimplantation (±psoas hitch or Boari flap)	13 (52.0)
Ureteral replacement with ileal segment	1 (4.0)
Near-infrared fluorescence imaging, n (%)	
No	8 (32.0)
Yes	17 (68.0)
Length of hospital stay (days), median (IQR)	3 (1–5)
Serum creatinine at discharge (mg/dL), median (IQR)	1.09 (1.00–1.23)
eGFR at discharge (mL/min), median (IQR)	67.7 (58.7–81.6)
Serum creatinine at last follow-up visit (mg/dL), median (IQR)	1.03 (0.80–1.20)
eGFR at last follow-up visit (mL/min), median (IQR)	77.0 (63.7–93.9)
Overall recurrences, n (%)	8 (15.1)

IQR, interquartile range; eGFR, estimated glomerular filtration rate.

**Table 3:** Logistic regression model predicting ureteral stricture recurrence.

	OR	95% C.I.	*p*-Value
**Univariate analysis**			
Age	1.1	0.9–1.2	0.1
BMI	0.9	0.9–1.0	0.9
Stricture length > 2 cm	6.4	1.1–36.9	0.04
Total dose	1.0	0.9–1.1	0.3
Radiotherapy duration	1.5	0.9–2.5	0.1
Concomitant chemotherapy	8.9	1.6–49.9	0.01
**Multivariate analysis**			
Stricture length > 2 cm	5.6	0.9–36.4	0.07
Concomitant chemotherapy	7.8	1.3–49.0	0.03

OR, odds ratio; C.I., confidence interval; BMI, Body Mass Index.

## Data Availability

The datasets presented in this article are not readily available due to technical and time limitations.
